# Anther Dissection and Confocal Imaging of Developing and Mature Pollen in *Arabidopsis*

**DOI:** 10.21769/BioProtoc.5862

**Published:** 2026-11-05

**Authors:** Yuan Zhou, Anna A. Dobritsa

**Affiliations:** 1 Beijing Vegetable Research Center (BVRC), Beijing Academy of Agriculture and Forestry Sciences, Beijing, China; 2 Department of Molecular Genetics, Ohio State University, Columbus, OH, USA

**Keywords:** Pollen development, Pollen lineage cells, Confocal microscopy, Protein localization, Fluorescence quantification

## Abstract

Pollen development is a fundamental process underlying plant fertility that involves a series of coordinated cellular transitions from microspore mother cells (MMCs) to mature pollen grains. Conventional approaches for studying this process often require tissue sectioning, extensive sample preparation, and specialized instrumentation operated by trained personnel, making them labor-intensive, time-consuming, costly, and poorly suited for high-throughput analysis. Here, we describe simpler protocols for preparing and imaging both developing pollen lineage cells and mature pollen grains from *Arabidopsis*. By combining targeted anther dissection with gentle extrusion of pollen cells from anthers, fluorescent staining, and confocal microscopy, our protocol makes pollen cells at different developmental stages accessible for high-resolution imaging, enabling visualization of cellular morphology and protein localization, as well as fluorescence-based protein quantification. We also describe a complementary workflow for imaging mature pollen grains that provides a rapid alternative to scanning electron microscopy (SEM) for examining pollen surface structures and patterns. Together, these protocols offer practical approaches for investigating the cellular and molecular processes underlying pollen development and for characterizing the surface morphology of mature pollen.

Key features

• Individual pollen lineage cells are released from *Arabidopsis* anthers for direct confocal imaging.

• Microspore mother cells (MMCs), dividing MMCs, tetrads, and free microspores are obtained within a single preparation, facilitating stage-to-stage comparisons.

• Protocol is compatible with multichannel confocal imaging, three-dimensional reconstruction, and fluorescence-based protein localization and quantification analyses.

• Protocol provides a rapid confocal microscopy–based alternative to SEM for examining pollen surface structures and patterns.

## Graphical overview


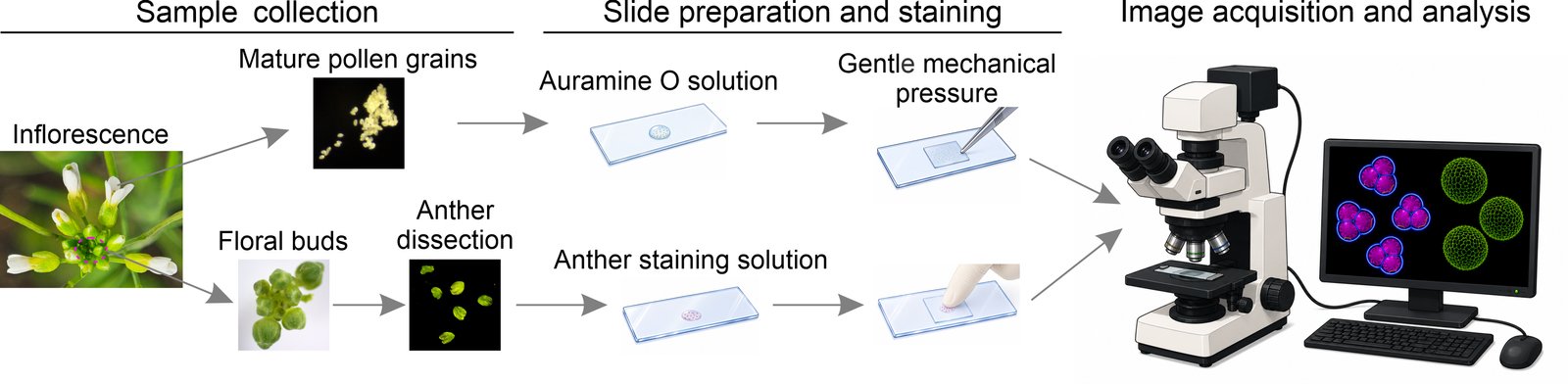


## Background

In flowering plants, pollen development, which is critical for male fertility and crop yield, is a highly orchestrated process. It involves meiotic division of microspore mother cells (MMCs), formation of tetrads of microspores enclosed within a common callose wall, release of free young microspores after the callose wall is degraded, synthesis of the pollen wall, formation of gametes, and maturation into functional pollen grains [1–3]. Pollen develops underneath multiple layers of anther tissues, which limits optical accessibility of pollen lineage cells and makes it challenging to visualize their intracellular structures, accurately determine the localization of molecules, and perform quantitative analyses [4–6]. Traditionally, pollen development has been characterized using ultrathin sectioning followed by transmission electron microscopy (TEM) [7–10]. While TEM offers excellent structural resolution, it is labor-intensive, time-consuming, and not well-suited for the high-throughput analysis of protein expression and localization. For mature pollen grains, scanning electron microscopy (SEM), commonly used to examine pollen surface structures such as exine and apertures, also requires relatively complex sample preparation and is low throughput [11–13].

The protocols described here provide rapid and accessible alternatives to TEM and SEM by combining simple sample preparations of developing anthers or mature pollen with confocal microscopy. Fluorescent staining of mature pollen grains with Auramine O allows visualization of exine structures and aperture patterns across the entire pollen surface at high resolution [14,15]. Preparation of developing anthers allows imaging of pollen lineage cells, including MMCs, dividing MMCs, tetrads, and free microspores, both within anthers and released from anthers. Imaging of developing pollen inside anthers can be used, for example, to rapidly screen transgenic lines for protein expression, accurately determine the pollen’s developmental stage, visualize other anther cell layers, or compare pollen development and protein expression and localization among the four locules of the same anther or among developing pollen grains within a single locule. Pollen lineage cells released from anthers are suitable for high-resolution z-stack imaging and three-dimensional reconstruction [16–18]. Consequently, protein localization, particularly at the cell surface, can be visualized more clearly and accurately than in intact anther tissues [5,18]. Beyond *Arabidopsis*, these protocols can be adapted and optimized for other flowering plant species [19–22], facilitating broader applications in the study of pollen development and pollen wall patterning.

## Materials and reagents

**Biological materials**

1. 5–7-week-old flowering *Arabidopsis thaliana* plants

*Note:* A. thaliana *plants can be wild type, mutants, or transgenic lines expressing fluorescently tagged proteins of interest for protein localization studies and quantification of expression levels. Plants are grown in soil at 20–22 °C under a 16/8 h light/dark cycle in growth chambers or in a greenhouse. For imaging anthers and developing pollen, younger plants shortly after the onset of flowering provide the best samples. For imaging mature pollen, plants of any age can be used, as long as they still flower.*

**Reagents**

1. Auramine O (Sigma-Aldrich, catalog number: A9655-25G, CAS number: 2465-27-2) (to stain the exine)

2. Tris base (Fisher Scientific, catalog number: BP152-1, CAS number: 77-86-1)

3. VECTASHIELD antifade mounting medium (Vector Laboratories, catalog number: VEC-H-1000)

4. Calcofluor White (also called Fluorescent Brightener 28) (Sigma, catalog number: F3543, CAS number: 4404-43-7) (to stain callose wall, primexine, and intine in pollen lineage cells, as well as cellulose in cell walls of other anther cell layers)

5. CellMask^TM^ Deep Red plasma membrane stain (Invitrogen^TM^, catalog number: C10046) (to stain the plasma membrane and endomembranes of all anther cells)

6. Dimethyl sulfoxide (DMSO) (Sigma, catalog number: D8418, CAS number: 67-68-5)

**Solutions**

1. 100× Auramine O working solution (see Recipes)

2. 10× CellMask^TM^ Deep Red (see Recipes)

3. 2% Calcofluor White (see Recipes)

4. Anther staining solution (see Recipes)

**Recipes**

**1. 100× Auramine O stock solution**

**Table:** 

Reagent	Final concentration	Quantity or volume
Auramine O	0.1%	10 mg
50 mM Tris-HCl (pH 7.5)	n/a	10 mL
Total	n/a	10 mL

*Note: Auramine O is difficult to dissolve completely, and it is normal to see some precipitate. The stock solution should be protected from light and is stable for up to 6 months at room temperature. To prepare the working solution, dilute a freshly prepared stock solution 100-fold with distilled water and keep it at room temperature, protected from light. If using an older stock solution, a lower dilution (e.g., 50-fold) may be required to compensate for reduced staining intensity and obtain a pale-yellow working solution.*

**2. 10× CellMask^TM^ Deep Red**

**Table:** 

Reagent	Final concentration	Quantity or volume
5 mg/mL CellMask^TM^ Deep Red	50 μg/mL	1 μL
DMSO	n/a	99 μL
Total	n/a	100 μL

*Note: The 5 mg/mL CellMask^TM^ Deep Red concentrate should be stored at -20 °C and the 10× CellMask^TM^ Deep Red stock solution can be stored protected from light at 4 °C for long-term use. Both the concentrate and the 10× stock are highly stable when properly stored and can be kept for many years.*

**3. 2% Calcofluor White**

**Table:** 

Reagent	Final concentration	Quantity or volume
Calcofluor White	2% (m/v)	20 mg
Distilled H_2_O	n/a	1 mL
Total	n/a	1 mL

*Note: The 2% Calcofluor White stock solution can be stored at room temperature, but should be sealed with parafilm to prevent evaporation.*

**4. Anther staining solution**

**Table:** 

Reagent	Final concentration	Quantity or volume
VECTASHIELD antifade mounting medium	89% (v/v)	89 μL
10× CellMask^TM^ Deep Red (50 μg/mL)	5 μg/mL (1×)	10 μL
2% Calcofluor White	0.02% (v/v)	1 μL
Total	n/a	100 μL

*Note: This solution should be protected from light and stored at 4 °C for long-term use. Additional fluorescent dyes, such as the nuclear stain DAPI (final concentration, 5 μg/mL), may be added to the anther staining solution as needed.*

**Microscopy supplies**

1. Standard microscope slides (3 × 1 in, 1 mm thick) (Fisher Scientific, catalog number: 12-550-123, or similar)

2. Microscope coverslips, 18 × 18 mm, #1, thickness 0.13–0.16 mm (Kemtech, catalog number: 0340-0150, or similar)

3. Nail polish (Electron Microscopy Sciences, catalog number: 72180, but different brands of clear nail polish from local pharmacies work just as well)

4. Type A immersion oil (Electron Microscopy Sciences, catalog number: 50-266-46)

## Equipment

1. Tweezers (World Precision Instruments Dumont #5, 11 cm, 0.05 × 0.01 mm tips, Fisher Scientific, catalog numbers: 50-822-450 and 50-822-205)

2. Insect pins and minuten pins mounted in a pin vise (fine insect pins: Austerlitz insect pins, size 000, Entomoravia, or similar; very fine minuten pins: Fine Science Tools, catalog number: 26002-10; Starrett pin vise #240-A: Grainger, catalog number: 2ZVH5)

*Note: Insect and minuten pins are highly recommended for anther dissection, as they are thinner, less expensive, and remain sharp much longer than tweezers. Minuten pins can also be bent into a small hook when needed. Both insect pins and minuten pins fit in the Starrett pin vise #240-A. To provide a more comfortable handle, we insert the pin vise into the barrel of an empty BIC pen after removing the ink reservoir with pliers and secure it with labeling tape (Figure 1).*

**Figure 1.**
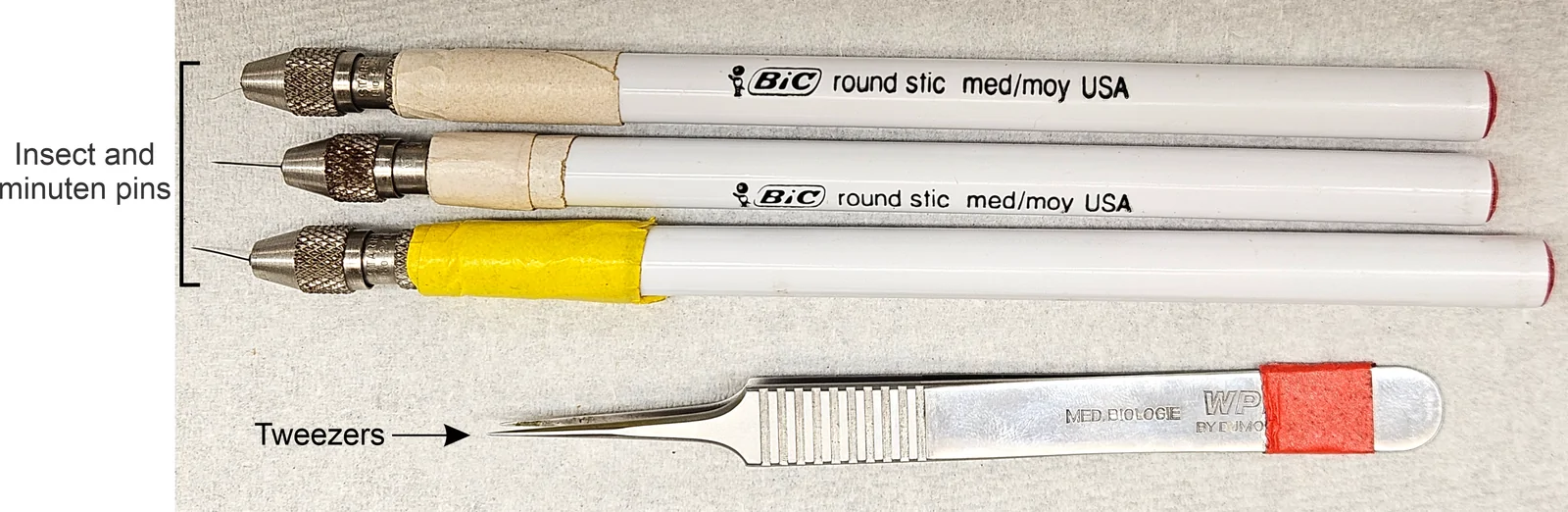
Dissection tools used in this protocol

3. Horizontal cardboard slide holders (USP, catalog number: 96080 or similar)

4. Dissecting microscope (Nikon, model: SMZ745 or similar)

5. Nikon A1+ confocal microscope or another confocal microscope

## Software and datasets

1. NIS-Elements imaging software (Nikon, v. AR 5.41.02)

*Note: This is commercial software that provides tools for image acquisition, analysis, and 3D visualization. A free version, NIS-Elements Viewer, can be used to view the images (not currently available for MacOS). Alternatively, open-access NIH ImageJ/Fiji software can be used for image analysis.*

## Procedure

**A. Imaging mature pollen grains**

1. Prepare the Auramine O working solution according to the recipe described above.

*Note: In our experience, the Auramine O stock solution can be prepared in advance and stored at room temperature in the dark for up to 6 months; the working solution can be stored at room temperature protected from light for up to 2 months. The stock solution is bright yellow, and the working solution is pale yellow (Figure 2A).*

2. Bring *Arabidopsis* plants from the growth chamber or greenhouse to the microscopy room and place them near a dissecting microscope for convenient sample collection.

3. Label a microscope slide with the sample name and date.

4. Select an open flower from the plant of interest and remove it with tweezers.

*Note: Using flowers that have opened on the day of pollen collection is recommended as they provide the highest amount of pollen. However, some pollen can typically be collected from any open flower.*

5. Hold the flower at its base with tweezers and invert it over the slide. Gently tap the flower against the slide to release pollen grains. Alternatively, place the flower directly onto the slide and gently pat the anthers with tweezers or a pin to release pollen grains.

*Notes:*

*1. We often use pollen from 2–4 flowers of the same plant in a single preparation to increase the number of pollen grains on the slide.*

*2. Removing petals is generally not required. They can be pushed out of the way with tweezers or a pin.*

*3. When preparing and visualizing pollen samples under a dissecting microscope, we use the highest available magnification (75× in our setup: 10× eye pieces; 1.5× objective; 5× zoom).*

6. With an insect pin, remove any debris present in the vicinity of the pollen.

7. Pipette 5–6 μL of Auramine O working solution directly onto the pollen grains. Gently spread the solution with a pin to ensure all pollen grains are fully immersed.

*Note: You can adjust the volume of Auramine O working solution according to the number of pollen grains on the slide or the size of the coverslip.*

8. Allow the pollen grains to hydrate in Auramine O working solution for approximately 5–10 min.

*Note: This incubation also allows much of the solution to evaporate, reducing the volume of liquid that will be spread beneath a coverslip. The incubation time may vary depending on the initial volume of Auramine O working solution and the evaporation rate. Monitor the slide during incubation and avoid complete evaporation of Auramine O working solution. If the solution evaporates completely, pollen grains may collapse, making it difficult to obtain clear images of pollen surface structures.*

9. Place a coverslip over the pollen grains, firmly press the coverslip using tweezers or a gloved finger at the center of the slide, and seal the edges with nail polish.

**Critical:** Proper sealing is essential to prevent dehydration of pollen grains during storage and confocal imaging.

*Notes:*

*1. We find that the best preparations tend to be on slides in which only a small amount of solution remains before a coverslip is placed. Gently tilt the slide to assess the amount of remaining solution. The slide is generally ready for coverslip placement when pollen grains no longer move freely with the solution, and only a thin layer of liquid remains. This also helps prevent the coverslip from slipping during sealing.*

*2. Applying pressure on the coverslip spreads the Auramine O solution into a thin layer and flattens pollen grains, exposing a larger portion of their surface, which is better for confocal imaging. To achieve optimal results, use the smallest amount of solution that can be fully spread beneath the entire coverslip. If the solution does not fully cover the space under the coverslip, more solution can be carefully added with a pipette before sealing.*

*3. Note that the amount of pressure applied to a coverslip should be determined empirically. The required amount of pressure can vary between samples and may need to be considerable (while avoiding breakage of the coverslip). If pollen does not appear fully flattened, additional pressure can be applied after the sample has been sealed. However, too much pressure could crush and rupture pollen grains. If pollen grains remain insufficiently flattened or become crushed, the sample should be prepared again.*

*4. When using 18* × *18 mm coverslips, two individual samples can be placed on the same slide under two coverslips (Figure 2B). This is convenient when processing large numbers of samples. Avoid placing the samples too close to the ends of the slide, where it will be difficult to image them.*

*5. Make sure to wipe the pins with tissue paper between samples to prevent inter-sample contamination.*

**Figure 2.**
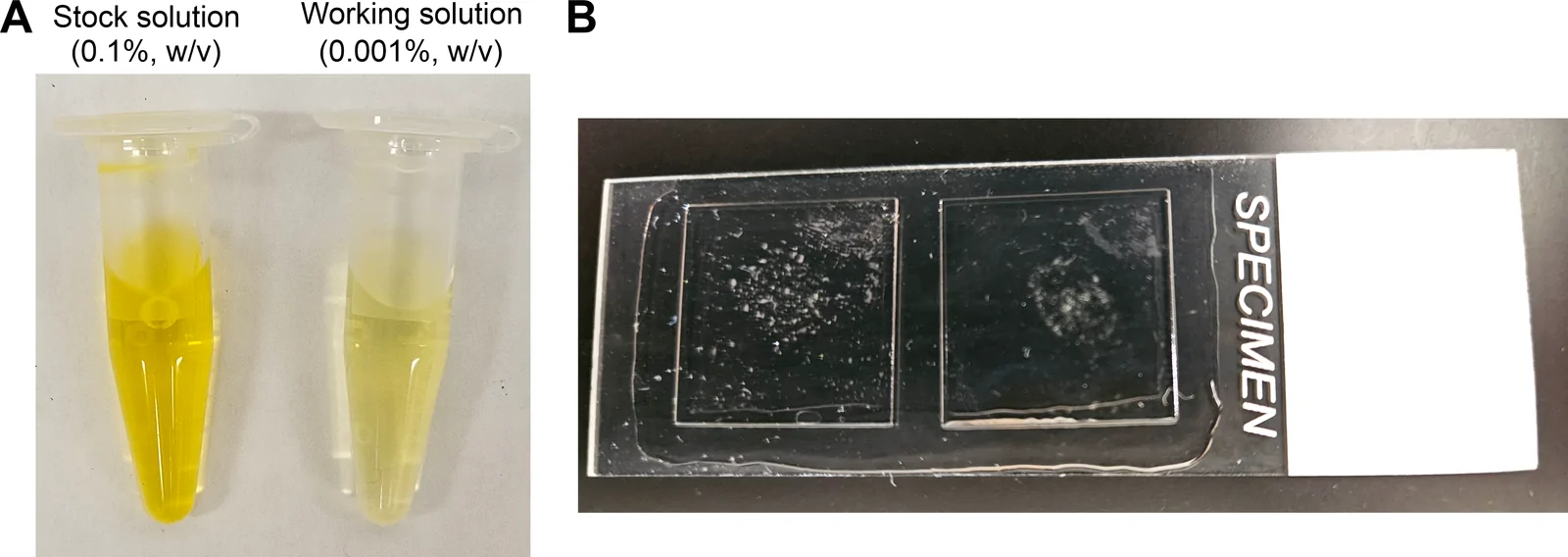
Examples of stock and working solutions of Auramine O and of two pollen samples mounted side by side on a single microscope slide. (A) Auramine O stock solution (0.1%, w/v; left) and working solution (0.001%, w/v; right). The stock solution is bright yellow, whereas the working solution is pale yellow. (B) Using two 18 × 18 mm coverslips allows two independent pollen samples to be prepared on the same slide, increasing sample throughput while reducing slide consumption.

**Pause point:** Sealed slides can be stored in horizontal slide holders at 4 °C for up to two weeks without noticeable deterioration in image quality if the samples remain hydrated.

10. Examine the slide using a confocal laser scanning microscope (we use a Nikon A1+ inverted microscope). Users should receive appropriate training before operating a confocal microscope. Locate pollen grains using a low-magnification objective and then switch to a 100× oil-immersion objective (NA = 1.4).

11. Use a GFP filter to observe pollen and select morphologically intact, fully hydrated, and flattened pollen grains for imaging (Figure 3). Adjust the focal plane to visualize the pollen surface or other regions of interest. When imaging exine patterns, make sure to focus on the surface (i.e., tectum) (Figure 3) and not on the baculae below. Adjust the detector high voltage (HV)/gain settings according to the fluorescence intensity of the sample to avoid over- and undersaturation.

12. Acquire images using 488-nm excitation and collecting emission between 500 and 550 nm. A zoom factor of 3× and a resolution of 512 × 512 pixels are recommended for imaging individual *Arabidopsis* pollen grains.

*Notes:*

*1. Well-flattened* Arabidopsis *pollen typically requires 10–15 z-steps at 500-nm intervals to span the distance between the front and back surfaces. Pollen that is not adequately flattened typically requires ~25 z-steps to span this distance.*

*2. For aperture quantification, taking images from both the front and back sides of the pollen grain is recommended to ensure accurate counting of all apertures (Figure 3).*

*3. Under identical imaging settings, fluorescence intensity is often higher on the front side of pollen grains (i.e., closer to the objective) than on the back side due to differences in imaging depth. Therefore, for z-stack acquisition and 3D reconstruction, HV/gain settings should be adjusted at different focal depths to maintain approximately uniform signal intensity throughout the z-axis and avoid excessive brightness near the front surface and weak signals near the back surface. The programmable z-intensity correction available in some confocal software packages is particularly convenient for this purpose.*

*4. To obtain the best-quality images of pollen exine, we recommend using the averaging function during image acquisition. We typically use 8× or 16× line averaging when imaging exine patterns.*

**Figure 3.**
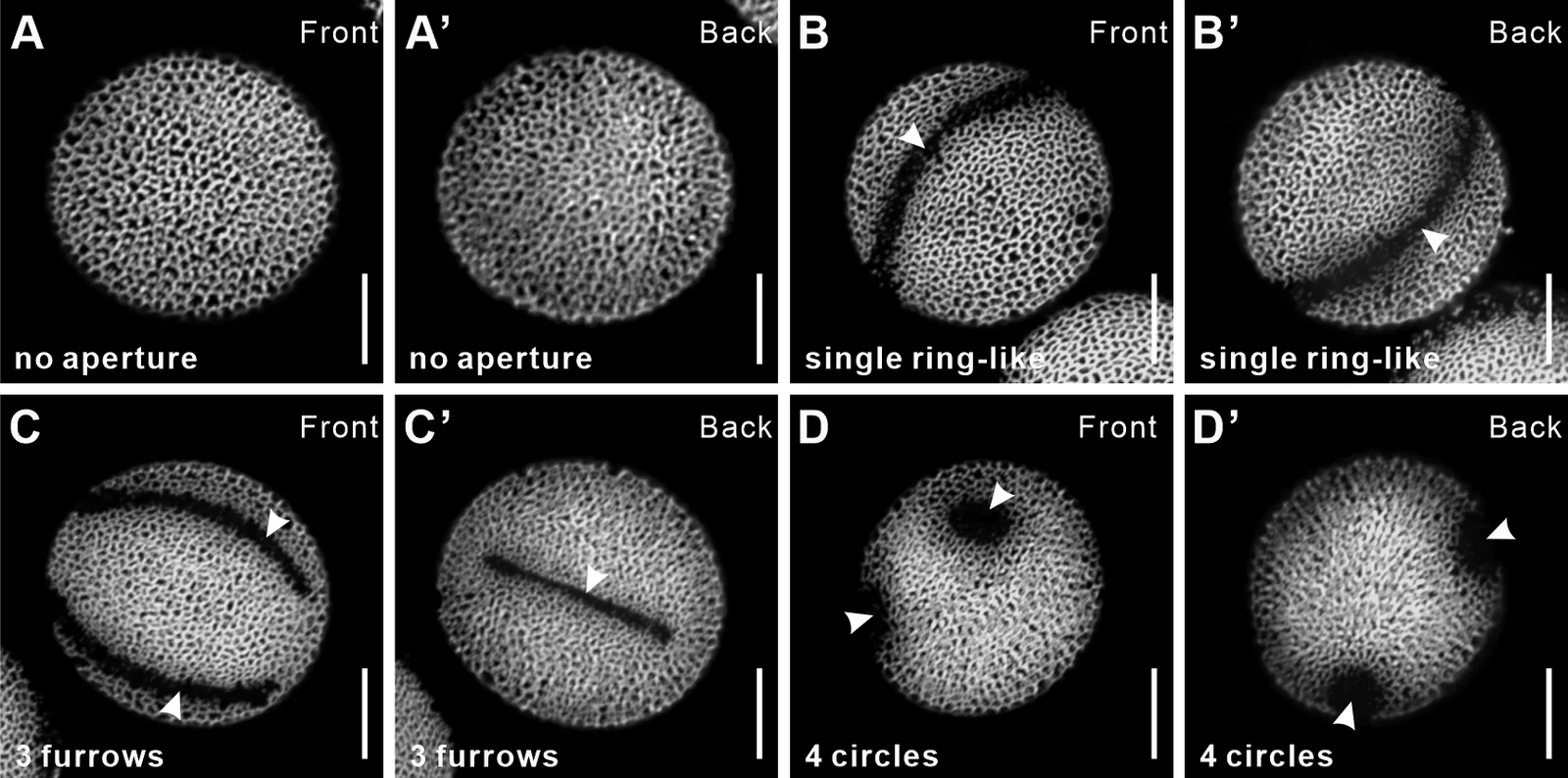
Examples of well-flattened *Arabidopsis* pollen grains that allow excellent views of reticulate exine and aperture patterns on the pollen surface. Pairs of images with the same letter (e.g., A and A’) show the front and the back views of the same pollen grain. Pollen grains with four different aperture patterns are shown. Apertures are indicated with arrowheads. Scale bars, 10 μm. All images were acquired using a 488 nm laser at 4% laser power and 3× confocal zoom, while the high voltage (HV)/gain was adjusted individually for each image to optimize visualization of pollen surface morphology and ranged from 75 to 94.

**B. Imaging developing pollen lineage cells**

1. Prepare the anther staining solution (see Recipes).

*Note: The staining solution can be prepared in advance and stored at 4 °C for long-term use.*

2. Bring *Arabidopsis* plants from the growth chamber or greenhouse to the microscopy room and place them near a dissecting microscope for convenient sample collection.

3. Using tweezers, carefully excise an inflorescence containing unopened flower buds from a healthy *Arabidopsis* plant (Figure 4A, C) and place it on a microscope slide away from the center of the slide.

4. Under a dissecting microscope, separate flower buds using an insect pin or a minuten pin mounted in a pin vise. We typically use an insect pin held in the non-dominant hand to keep the flower buds in place and a second insect pin or a minuten pin in the dominant hand for dissection and finer manipulations. Minuten pins can be made into a hook if you find it convenient for dissection.

*Note: Large flower buds containing older pollen stages can be removed with tweezers before dissection. Very small buds contain anthers that are too young and are usually not used during the preparation of developing pollen lineage cells.*

5. Select 3–4 flower buds corresponding approximately to floral stages 9–10 [23] (Figure 4) and remove or open their sepals using insect or minuten pins under a dissecting microscope. Petals are still very small at this stage and do not need to be removed. We find a 60× magnification of the dissecting microscope to be optimal for dissection (in our setup, a combination of 10× eyepieces, 1.5× objective, and 4× zoom).

*Note: Buds at the floral stages 9–10 can be identified according to [23] and correspond to anther stages 5–9 that contain pollen from MMC to young microspores beginning to develop exine [1]. Each inflorescence typically contains 3–4 buds at these stages (Figure 4).*

**Figure 4.**
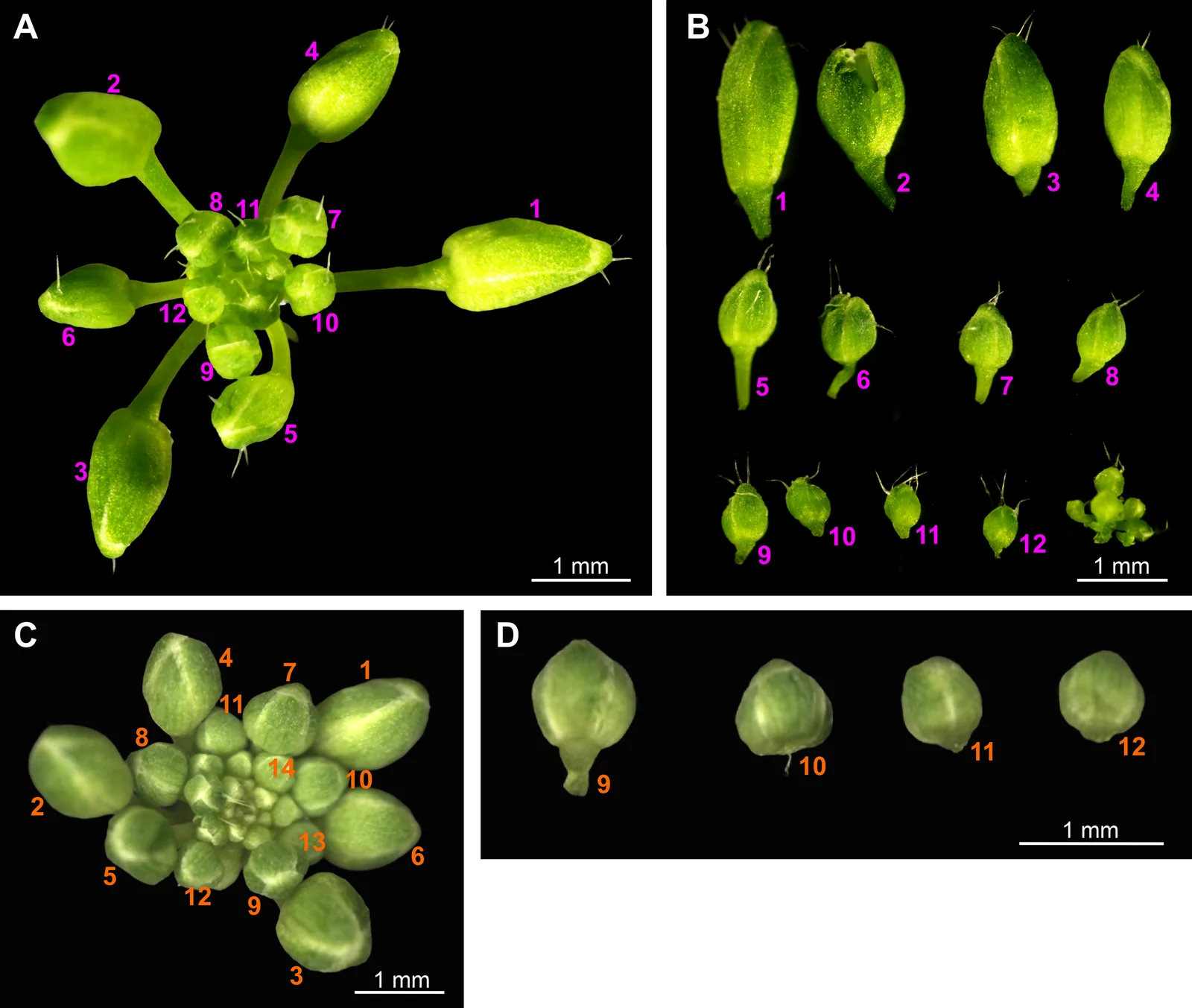
Selection of flower buds for imaging developing pollen lineage cells. (A) Representative *Arabidopsis* Columbia (Col-0) inflorescence with unopened flower buds numbered from oldest (1) to youngest (12). (B) Corresponding dissected Col-0 flower buds, arranged in the same order. Buds 9–12 are typically at floral stages 9–10 and are recommended for this protocol. Buds younger than 12 were not dissected. (C) Representative *Arabidopsis* Landsberg *erecta* (Ler) inflorescence with unopened flower buds numbered from oldest (1) to youngest (14). (D) Dissected Ler flower buds corresponding to the floral stages 9–10. Scale bars, 1 mm.

6. Using a pin, carefully remove anthers from each flower bud and transfer them into a 3 μL drop of anther staining solution placed at the center of the microscope slide.

*Notes:*

*1. We typically dissect at least 6–8 buds from a minimum of 2 independent inflorescences in a single preparation. Fewer buds may yield an insufficient number of cells at the desired developmental stages, whereas too many buds may result in cell overlap, compromising single-cell imaging.*

*2. When very accurate staging is required, each sample should contain anthers from a single bud.*

*3. Although the exact volume of anther staining solution is not critical, we recommend starting with 3–4 μL, which is usually sufficient to cover the sample after mounting. Larger volumes may allow cells to drift during imaging, making it difficult to acquire high-quality images.*

7. Use tissue paper to wipe debris of the dissected inflorescences from the slide. The same slide can be cleaned and reused for subsequent dissections.

8. With a pin, distribute the dissected anthers evenly in the staining solution and place a coverslip over the sample.

9. While observing the sample under the dissecting microscope, apply pressure with a gloved thumb to the coverslip directly over the anthers to release developing pollen lineage cells.

**Critical:** Apply only moderate pressure: less than needed for flattening mature pollen. Excessive compression may damage cells and compromise image quality.

*Notes:*

*1. We typically press the coverslip gently, then examine the slide under a dissecting microscope to assess cell release. If cells are not released, apply additional gentle pressure and recheck.*

*2. Sealing with nail polish is not required in anther preparations because the anther staining solution is sufficiently viscous to minimize evaporation. Unsealed slides can be stored horizontally at 4 °C for a maximum of one week. However, for protein localization and expression analysis, imaging should be completed within 2–3 days.*

*3. If the anther staining solution does not adequately cover the sample after pressing on the coverslip, a small additional amount of solution can be carefully added under the coverslip.*

10. Examine the slide using a confocal laser scanning microscope (we use Nikon A1+). Locate the anthers and released cells using a low-magnification objective and then switch to a 100× oil-immersion objective (NA = 1.4).

11. Use the following excitation and emission settings:

a. Calcofluor White: 405 nm excitation, 424–475 nm emission.

b. CellMask^TM^ Deep Red: 640 nm excitation, 663–738 nm emission.

c. GFP: 488 nm excitation, 500–550 nm emission.

d. YFP and other yellow fluorophores: 514 nm excitation, 522–555 nm emission.

e. RFP, mCherry, and other red fluorophores: 561 nm excitation, 580–630 nm emission.

12. Scan the slide to identify anthers and pollen lineage cells at different developmental stages, such as MMCs, dividing MMCs, tetrads, and free microspores (Figure 5). Select appropriate optical sections and acquire images at a resolution of 512 × 512 pixels using 1× zoom for anthers, 5× zoom for MMCs and tetrads, and 5× or 8× zoom for free microspores.

**Critical:** Carefully adjust HV/gain settings to avoid signal saturation. For comparisons of protein expression, identical imaging settings should be maintained across all compared samples.

*Note: To simultaneously visualize callose walls, plasma membranes, and fluorescent proteins, multichannel imaging is recommended.*

13. When acquiring z-stack images, a step size of 500 nm produces good results. For tetrads, this typically leads to 30–35 optical sections.

*Note: Given the significant amount of time required for acquiring z-stack images of tetrads (~1–2 min/tetrad), we usually do not use the averaging function. However, if better image quality is desired, 2× line averaging can be performed.*

14. Generate three-dimensional reconstructions using an imaging analysis software to visualize cell morphology, as well as the spatial distribution and cell-surface localization patterns of fluorescently labeled proteins (Figure 5C).

**C. Quantification of fluorescence intensity during pollen development**

1. Open the images selected for comparison in the NIS-Elements imaging software or another image analysis software.

*Notes:*

*1. Only images acquired using identical imaging settings should be included in fluorescence intensity comparisons.*

*2. For multichannel images, protein fluorescence quantification should be performed using only the channel corresponding to the fluorescent reporter. For example, when multiple channels are acquired (e.g., CellMask^TM^ Deep Red, Calcofluor White, and YFP), only the YFP channel should be used for analysis of protein fluorescence intensity.*

2. Use the region of interest (ROI) tool to define the region to be quantified by manually outlining it.

*Note: The size and location of ROIs depend on the biological question being addressed. Depending on the protein localization pattern, ROIs may include an entire cell, plasma membrane region, an aperture membrane domain, or other subcellular regions of interest. ROI selection criteria should be applied consistently across all samples being compared.*

3. Open the *Measurements* module and select the parameters for analysis.

*Note: We typically record ROI ID, ROI area, ROI mean intensity, ROI min intensity, ROI max intensity, and ROI sum intensity.*

4. Perform ROI-based measurements and export the results as an Excel or CSV file for downstream analysis.

*Notes:*

*1. Measure background fluorescence from a region lacking a specific fluorescence signal and use this value for background correction, when appropriate.*

*2. For each genotype, measure the fluorescence intensity of at least 30 pollen lineage cells.*

5. Perform statistical analyses using methods appropriate to the experimental design.

**Figure 5.**
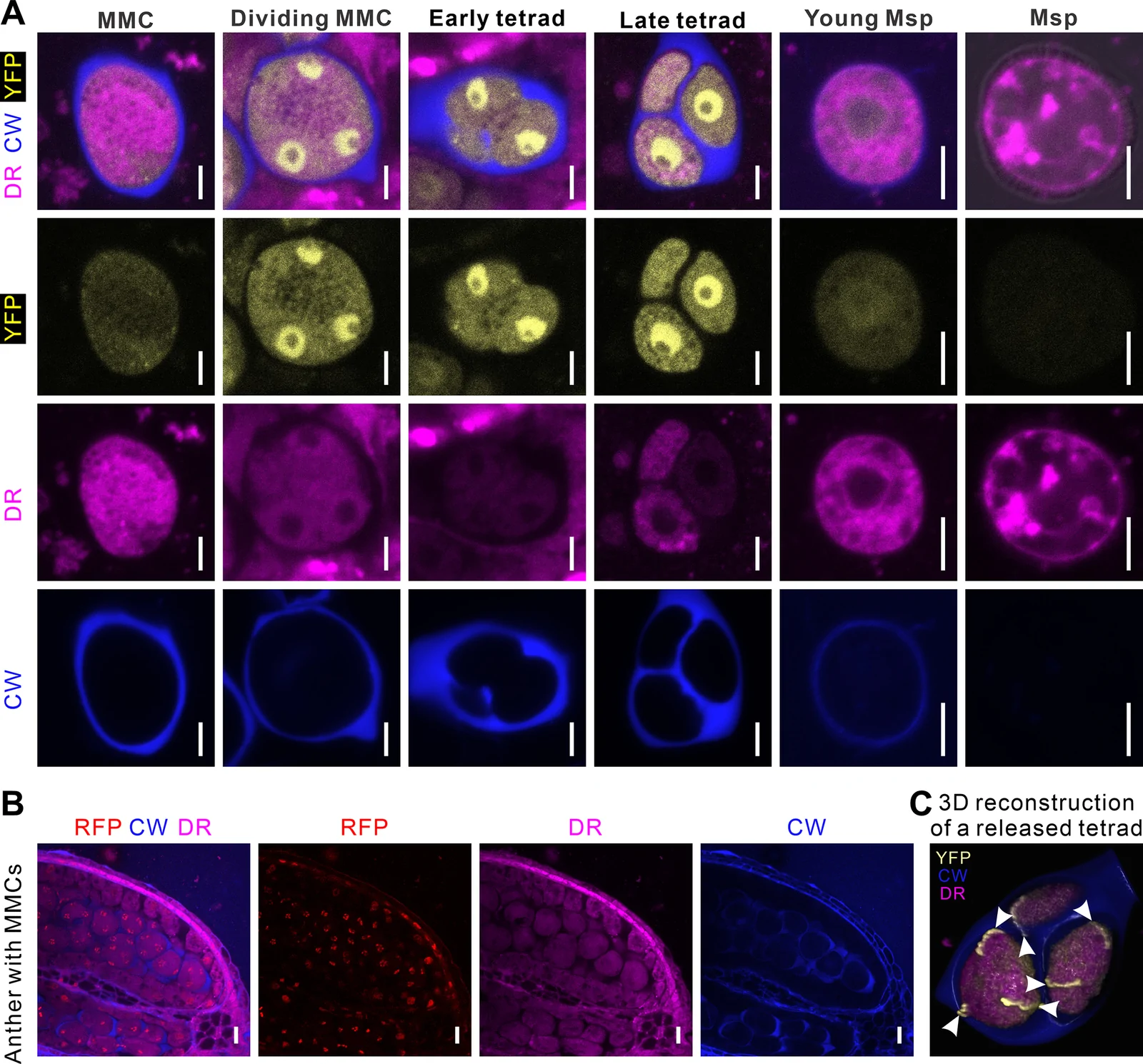
Examples of developing pollen lineage cells released from anthers, an intact anther containing microspore mother cells (MMCs) undergoing meiosis, and a three-dimensional reconstruction of a tetrad of microspores. (A) Representative confocal images of an MMC, dividing MMC, early tetrad, late tetrad, free young microspore (Msp), and mature microspore released from anthers. These pollen lineage cells express a YFP-tagged protein (YFP, yellow) and are stained with CellMask^TM^ Deep Red (DR, magenta), which stains membranous structures, and Calcofluor White (CW, blue), which stains the callose walls of MMCs and tetrads and the primexine of young microspores. Scale bars, 5 μm. (B) Confocal images of an intact anther containing MMCs undergoing meiosis, with chromosomes labeled with histone H2B-RFP (red). The anther is stained with DR (magenta) and CW (blue). Here, CW stains not only the callose walls of MMCs (located in the middle of anther locules) but also the cell walls of several other anther layers. Scale bars, 10 μm. (C) 3D reconstruction of a tetrad released from an anther. Arrowheads indicate the YFP-tagged protein accumulating at the aperture domains on the surface of each microspore. The tetrad is also stained with DR (magenta) and CW (blue).

## Validation of protocol

This protocol (or parts of it) has been used and validated in the following research articles:

• Zhou et al. [16]. Members of the ELMOD protein family specify formation of distinct aperture domains on the *Arabidopsis* pollen surface. *eLife* (Figures 1–7, 9, and 10).

• Zhou et al. [24]. A trio of *Arabidopsis* proteins acts as the executor complex in the pollen aperture formation program. *Plant Cell* (Figures 1, 3–5, and 8).

• Lee et al. [25]. A species-specific functional module controls formation of pollen apertures. *Nat Plants* (Figures 1–3, 7, and 8).

• Plourde et al. [26]. Changes in morphogen kinetics and pollen grain size are potential mechanisms of aberrant pollen aperture patterning in previously observed and novel mutants of *Arabidopsis thaliana. PLoS Comput Biol* (Figures 1, 8, and 9).

• Lee et al. [17]. *Arabidopsis* protein kinase D6PKL3 is involved in the formation of distinct plasma membrane aperture domains on the pollen surface. *Plant Cell* (Figures 1–10).

• Li et al. [27]. INP1 involvement in pollen aperture formation is evolutionarily conserved and may require species-specific partners. *J Exp Bot* (Figures 2 and 4–6).

• Dobritsa et al. [18]. Pollen aperture factor INP1 acts late in aperture formation by excluding specific membrane domains from exine deposition. *Plant Physiol* (Figures 1–5 and 7).

• Reeder et al. [5]. A ploidy-sensitive mechanism regulates aperture formation on the *Arabidopsis* pollen surface and guides localization of the aperture factor INP1. *PLoS Genet* (Figures 1–10).

## General notes and troubleshooting

**General notes**

1. These protocols were developed for *Arabidopsis thaliana* and have been successfully adapted to other flowering plant species (like rice, tomato, and California poppy) [19,20,22,27]. While minor adjustments in sample preparation and imaging conditions are necessary, the general procedures for isolating, imaging, and analyzing developing pollen lineage cells and mature pollen grains appear broadly applicable across species.

2. The developmental stage of selected floral buds determines the types of pollen lineage cells present in a preparation. In our experience, analyzing multiple floral buds from several (4–6) independent inflorescences provides sufficient cell numbers and increases the likelihood of obtaining MMCs, tetrads, and free microspores in the same sample.

3. Releasing developing pollen lineage cells from anthers requires applying an appropriate amount of pressure on the coverslip. If too little pressure is applied, few cells will be released; if too much pressure is applied, cells may be damaged. In most cases, gentle pressure is sufficient, and additional pressure can be applied gradually if needed. A similar principle applies to flattening mature pollen grains, although this typically requires greater pressure than that needed for anther release.

4. For fluorescence imaging and quantitative analyses, imaging parameters should be optimized to maximize signal detection while avoiding pixel saturation. When fluorescence intensity is compared among genotypes or treatments, all samples must be imaged using identical acquisition settings.

**Troubleshooting**

**Problem 1:** Mature pollen grains appear collapsed (Figure 6A–A’’).

Possible cause: Although collapsed pollen grains may be a phenotype of abnormal pollen development, they can also be caused by improper sample preparation. This happens when pollen grains are insufficiently hydrated or the Auramine O staining solution evaporates before mounting.

Solution: Increase the hydration time and ensure that the staining solution does not evaporate before the coverslip is applied and sealed.

**Problem 2:** Mature pollen grains are weakly stained and difficult to visualize.

Possible cause: The Auramine O solution has lost activity due to prolonged storage or was prepared at an incorrect concentration.

Solution: Prepare a fresh working solution and/or stock solution. The stock solution should appear bright yellow, whereas the working solution should be pale yellow.

**Problem 3:** Only a small portion of the pollen surface can be visualized during imaging (Figure 6B).

Possible cause: Excess staining solution remained under the coverslip, preventing the pollen grains from being flattened and limiting the observable surface area.

Solution: Allow the drop of Auramine O solution containing hydrating pollen to evaporate substantially, but not completely, before placing the coverslip. After placing the coverslip, press firmly on it. If some excess staining solution is still present, remove it from the edge of the coverslip using tissue paper. In some cases, pressing firmly on a sealed coverslip of a sample in which pollen appears inadequately flattened can sufficiently flatten the pollen, eliminating the need to prepare a new sample (Figure 6C–C’’).

**Problem 4:** Mature pollen grains or developing pollen lineage cells appear ruptured (Figure 6D–E’).

Possible cause: Excessive pressure was applied during mounting.

Solution: Apply gentler pressure when mounting the sample. Examine the sample under the confocal microscope and apply additional pressure gradually if needed.

**Figure 6.**
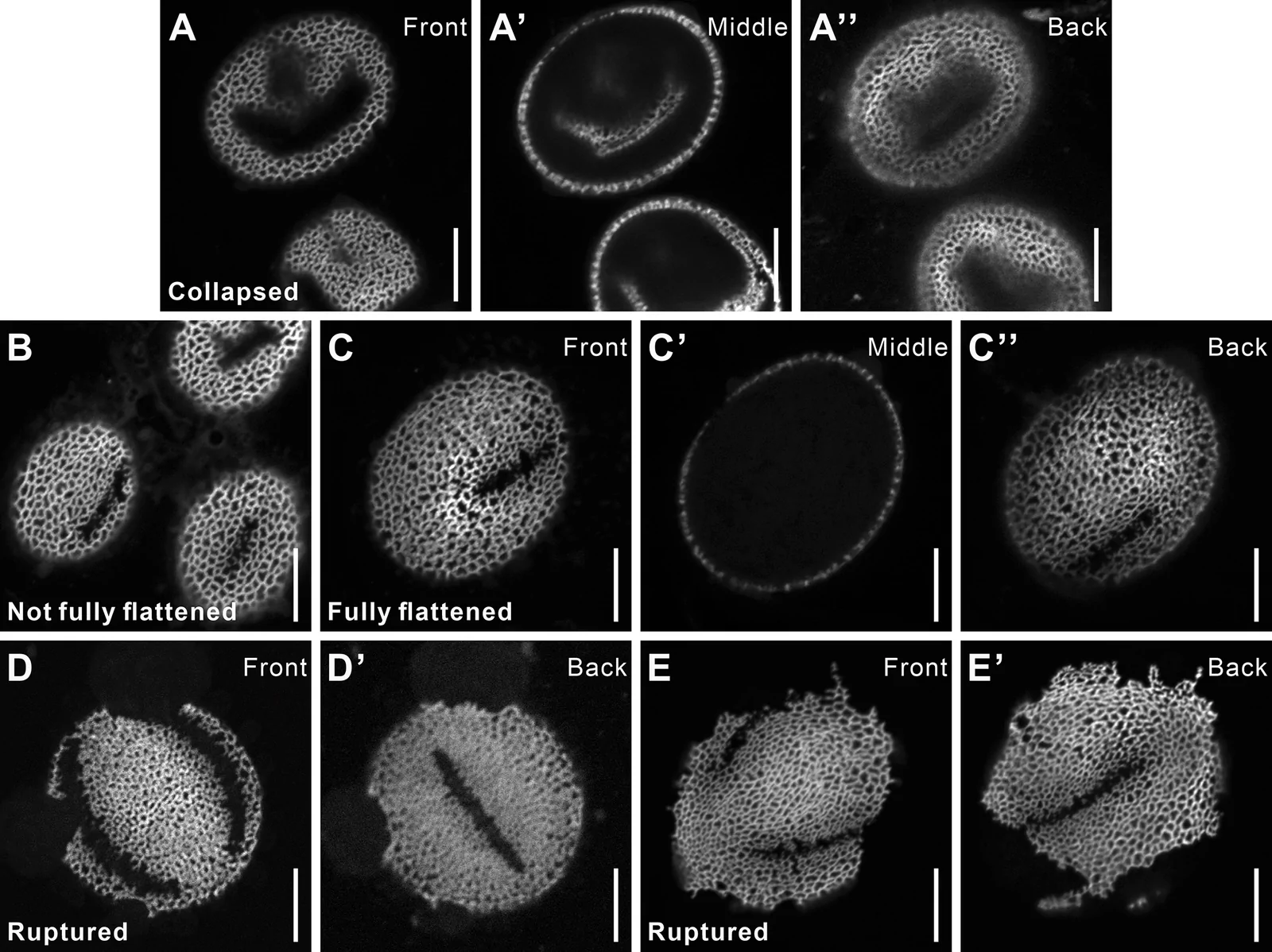
Examples of poor-quality preparations of mature pollen. (A–A’’) Collapsed pollen grain shown at the front, middle, and back focal planes. (B–C’’) Comparison of pollen grains that are not fully flattened (B) with a fully flattened one (C–C’’). Pollen grains appear substantially smaller when not fully flattened. (D–E’) Two examples of ruptured pollen grains resulting from excessive pressure on the coverslip. Scale bars, 10 μm.

**Problem 5:** Developing pollen lineage cells are crowded and difficult to image individually.

Possible cause: Insufficient anther staining solution was used, causing cells to cluster together after release.

Solution: Increase the volume of anther staining solution (approximately 3–4 μL is recommended as a starting point).

**Problem 6:** Few or no developing pollen lineage cells are released from the anthers.

Possible cause: Insufficient pressure was applied to the coverslip.

Solution: Apply additional gentle pressure to the coverslip and re-examine the sample.

**Problem 7:** The desired developmental stage is not observed.

Possible cause: Flower buds were collected at inappropriate developmental stages.

Solution: Adjust bud selection according to the developmental stage of interest. In older plants, inflorescences may lack buds containing some anther stages—in particular, tetrads. For this reason, it is recommended to use plants that have recently begun flowering.

**Problem 8:** Developing pollen lineage cells move during imaging.

Possible cause: Excessive anther staining solution was used, allowing cells to drift under the coverslip.

Solution: Reduce the volume of anther staining solution used for sample preparation.

**Problem 9:** Fluorescence signals are weak.

Possible cause: Staining reagents have lost activity, staining concentrations are incorrect, or the fluorescent reporter is expressed at a low level.

Solution: Verify the activity and concentration of staining reagents. For fluorescent protein imaging, optimize acquisition settings and consider using transgenic lines with stronger reporter expression if signals remain weak.
